# Performance Evaluation of Vegetable Oil-Based Nano-Cutting Fluids in Environmentally Friendly Machining of Inconel-800 Alloy

**DOI:** 10.3390/ma12172792

**Published:** 2019-08-30

**Authors:** Munish Kumar Gupta, Muhammad Jamil, Xiaojuan Wang, Qinghua Song, Zhanqiang Liu, Mozammel Mia, Hussein Hegab, Aqib Mashood Khan, Alberto Garcia Collado, Catalin Iulian Pruncu, G.M. Shah Imran

**Affiliations:** 1 Key Laboratory of High Efficiency and Clean Mechanical Manufacture, Ministry of Education, School of Mechanical Engineering, Shandong University, Jinan 250000, China; 2 College of Mechanical and Electrical Engineering, Nanjing University of Aeronautics and Astronautics, Nanjing 210016, China; 3 National Demonstration Center for Experimental Mechanical Engineering Education, Shandong University, Jinan 250000, China; 4 Mechanical and Production Engineering, Ahsanullah University of Science and Technology, Dhaka 1208, Bangladesh; 5 Mechanical Design and Production Engineering Department, Cairo University, Giza 12163, Egypt; 6 Department of Mechanical and Mining Engineering, University of Jaen, EPS de Jaen, Campus Las Lagunillas, 23071 Jaen, Spain; 7 Mechanical Engineering, Imperial College London, Exhibition Rd., SW7 2AZ London, UK

**Keywords:** environmentally friendly, nano-cutting fluids, nickel-based alloys, turning, optimization

## Abstract

Recently, the application of nano-cutting fluids has gained much attention in the machining of nickel-based super alloys due their good lubricating/cooling properties including thermal conductivity, viscosity, and tribological characteristics. In this study, a set of turning experiments on new nickel-based alloy i.e., Inconel-800 alloy, was performed to explore the characteristics of different nano-cutting fluids (aluminum oxide (Al_2_O_3_), molybdenum disulfide (MoS_2_), and graphite) under minimum quantity lubrication (MQL) conditions. The performance of each nano-cutting fluid was deliberated in terms of machining characteristics such as surface roughness, cutting forces, and tool wear. Further, the data generated through experiments were statistically examined through Box Cox transformation, normal probability plots, and analysis of variance (ANOVA) tests. Then, an in-depth analysis of each process parameter was conducted through line plots and the results were compared with the existing literature. In the end, the composite desirability approach (CDA) was successfully implemented to determine the ideal machining parameters under different nano-cutting cooling conditions. The results demonstrate that the MoS_2_ and graphite-based nanofluids give promising results at high cutting speed values, but the overall performance of graphite-based nanofluids is better in terms of good lubrication and cooling properties. It is worth mentioning that the presence of small quantities of graphite in vegetable oil significantly improves the machining characteristics of Inconel-800 alloy as compared with the two other nanofluids.

## 1. Introduction

Nickel (Ni)-based alloys have become widely accepted materials for the manufacture of critical parts owing to their exceptional characteristics such as high creep, good rupture strength, and resistance to corrosion and oxidation [1]. Due to excellent fatigue strength and possession of yield strength at high temperature and pressure (600 °C, 1000 MPa), Ni alloys are used in the manufacturing of aero-engines, turbine blades, nuclear reactors, and in chemical industries, where there is a requirement for use of cyclic loads and high temperatures. Thakur and Gangopadhyay have examined an aero-engine consisting of 50% Ni alloy in weight, due its high thermal stability in severe environments [2]. In addition, Ni alloys are ductile materials under cryogenic temperature because of their face-centered cubic (FCC) structure, which is why they are used in cryogenic tanks, as superconducting materials, and in rocket motor casings [3]. Nowadays, Ni-based alloys have several grades, such as Inconel-718, FGH-95, ME-16, IN-100, Inconel-800, and Inconel-825. Among numerous Inconel grades, Inconel-800 is a Fe–Ni–Cr alloy that offers adequate resistance to oxidation, and carburization even at elevated temperatures with moderate strength [4]. It is highly desirable for the manufacturing of high temperature equipment which is resistant to chloride stress corrosion cracking and shows high creep and stress rupture characteristics in the temperature range of 594–983 °C. 

Despite all its advantages and applications, machining of such difficult-to-cut materials is also a great challenge due to their poor thermal conductivity, hot hardness, and chemical reaction with tool materials [5]. Such limitations have compelled the manufacturing industries to revise tool failure criteria for turning (ISO-3685) to attain adequate surface quality and tool life. Therefore, considerable attention has been dedicated to researching the manufacture aerospace components without compromising surface quality, in addition to tool edge damage. In the turning process, shearing and friction due to rubbing of chip at the tool rake face produces an elevated temperature in primary and secondary machining zones. This generated heat strongly effects the tool wear and surface quality because, above a certain temperature, tool binding may start losing its strength and accelerate wear. 

In order to reduce the temperature and acceleration of tool wear and to improve the surface quality, several lubri-cooling techniques have been practiced in industry. These coolants and lubricants remove chips and reduce temperature and friction due to the rubbing of chip and tool. Water-soluble oils and minerals oils are frequently applied in industry. However, due to their adverse effects on ecology, operator heath, and some restrictions from the EPA (Environmental Protection Agency), advanced industries have started accepting some sustainable cooling/lubrication techniques [6], such as minimum quantity lubrication (MQL) machining [7], cryogenic machining [8], and nanofluid-assisted MQL machining [9] in order to enhance the machinability of Inconel-800 alloy. In MQL machining, small quantities (microlubrication) of pure oil (vegetable oil) are mixed with compressed air to impinge a fine mist (10~100 mL) to attain the advantage of effective cooling and lubrication at the tool–chip interface. Most of research studies have shown better surface quality and tool life under MQL machining compared with dry or flood cooling [10,11]. Similarly, Gurraj et al. have investigated the machining of difficult-to-cut material under MQL to enhance machinability. Turning tests under the MQL lubri-cooling technique were carried out to improve the machinability in terms of surface quality, tool wear, and cutting forces. Findings have depicted a 15% improvement in all the responses under the MQL cooling technique [12]. Also, Joshi et al. [13] have investigated the turning of Incoloy-800 under dry, flood (600 L/h) and MQL (150 mL/h, 230 mL/h) cutting conditions from the perspective of surface quality and flank wear. The findings have depicted less wear and better surface quality under MQL conditions. However, MQL (230 mL/h) provided favorable results compared to MQL (150 mL/h) under all conditions. Maruda et al. [14,15] also studied MQL conditions. They claimed that MQL performs very well during the machining of different materials. From the above findings, it can be understood that although lower flow rates of MQL achieved better performance, they were nevertheless not suitable for machining due to the material being difficult to cut. The key reason behind this problem is due to the lower oil flow rate which fails to limit heat generation at primary and secondary cutting zones and evaporates immediately in the machining of difficult-to-cut materials.

In order to enhance the machinability performance of MQL, specifically for difficult-to-cut materials, several advancements in MQL have been applied in research, i.e., nanofluid-assisted MQL [16,17], hybrid nanofluid MQL [18], Ranque–Hilsch vortex tube [12], ionic liquid-assisted MQL (IL-MQL) [19], electrostatic MQL [20], vegetable oil-based solid lubricant MQL [21], and time-controlled MQL pulse [22]. Among these advancements of the MQL system, nanofluid-assisted machining of difficult-to-cut materials is a widely accepted alternative. In order to enhance the thermal characteristics of heat transfer in machining, different types nanoparticles are used with vegetable base oil, such as alumina (Al_2_O_3_), graphite, aluminum nitride (AlN), and molybdenum disulfide. The mentioned nanoparticles provide superior heat transfer, thermal conductivity, surface area, and Brownian motion. Considering the sustainable machining of difficult-to-cut materials, Khan et al. [23] carried out machining under conventional MQL and Al_2_O_3_ nanofluid-assisted MQL (NFMQL) from the viewpoint of temperature, surface roughness, and energy consumption. Findings have depicted the superiority of NFMQL with a 16%.2~34.5% reduction in temperature and 11.3%~12% reduction in surface roughness for all cutting conditions. It is mentioned that nanoadditives (size < 100 nm) have a biodegradable base oil-enhanced tribological behavior, owing to an amending effect, polishing, tribo-film formation, and ball bearing effect. The existence of nanoadditives in nanofluids enhance thermal conductivity, the heat transfer rate, and the nanoparticles deposited on the machining region behave as small bearings and fins, leading to heat dissipation and lubrication. This was proposed for industrial applications, where nanofluids have provided stability at the tool–chip interface for better surface quality due to a ball bearing effect. Padmni et al. [24] have applied molybdenum disulfide (MoS_2_) nanoparticle-based vegetable oil in conventional machining in order to improve the machinability from the perspective of surface roughness and tool wear. Results have underscored a maximum of a 37% reduction in tool wear and 44% reduction in surface roughness with 0.5 vol% nanoparticles in comparison with dry turning. Khan et al. [25] applied copper nano-additives (Cu-np)-based MQL in the conventional machining to improve the surface quality and machinability. Findings have depicted superior surface quality under nanofluid-assisted machining. They reported that the application of Cu-nps in biodegradable oil extended tribological film formation as well as thermal properties. Hence, Cu-np-assisted machining has minimized surface roughness and lowered the environmental impact.

According to the aforementioned state-of-art review, it is worth mentioning that the MQL system along with the nanofluid cooling conditions have been considered as a good alternative and help to significantly improve the machining performance in terms of lower cutting forces, surface roughness, tool wear, etc. Therefore, with this aim, the three type of nanofluids i.e., aluminum oxide (Al_2_O_3_), molybdenum disulfide (MoS_2_), and graphite with MQL system have been firstly implemented in the turning of new Inconel-800 alloy and various important characteristics such as cutting force, tool wear, and surface roughness were evaluated. Further, the process parameters were tested for their statistical significance levels using Box Cox transformation, normally distributed plots, and analysis of variance (ANOVA) methods, respectively. In the end, the optimized parameters were obtained using composite desirability approach (CDA). The paper is organized into the following sections (1) Introduction and Literature Review followed by (2) Materials and Methods, (3) results are presented in the Results and Discussion section and (4) the findings of complete paper are presented here.

## 2. Materials and Methods 

This section discusses the experimental setup used for machining of Inconel-800 alloy under nanofluid-enriched MQL conditions. The complete details of workpiece materials, mechanical properties, tool materials, and equipment used for machinability study are discussed below:

### 2.1. Workpiece, Cutting tool, and Machine Tool Details

In this work, the turning experiments were performed on new nickel-based alloy, i.e., Inconel-800. This alloy is mainly used in the aerospace, nuclear, and marine sectors. This alloy is used under heat-treated conditions. The chemical composition and heat treatment conditions of Inconel-800 alloy are presented in Table 1 and Table 2. The diameter and length of subjected material used was 50 mm × 120 mm, respectively. Further, the cutting tool used for machining the Inconel-800 alloy is cubic boron nitride (CBN) having model no. CCGW 09T304-2 tips and with rhombic shape. The insert was rigidly fixed on the tool holder of lathe tool dynamometer. The details of dynamometer are given in the following sections. Note that no separate tool holder is used for experimentation. This insert contains 50% of CBN content having a grain size of 2 µm, titanium carbide binder, and titanium nitride coating. Moreover, it is highly recommended to use interrupted cutting and heavy operations on high-strength temperature-resistant alloys, i.e., Inconel-800. The complete specifications of cutting tool are presented in Table 3. Further, the CNC lathe is used for performing the turning experiments on Inconel-800 alloy. This machine tool consists of two concurrently controlled axes, namely, Z axis (movement of carriage parallel to spindle axis (longitudinal)), and X-axis (movement of turret slide at right angle to spindle axis (cross)), and equipped with a Siemens control system. 

### 2.2. Cooling-Lubrication Conditions

Environmentally friendly cooling conditions, i.e., the minimum quantity lubrication system, have been implemented in this work. The MQL system used in this work was “*NOGA mini cool system’*. The main parts of this system are two pipes, nozzles, control valve, syphon line, and powerful on/off Popeye magnet system. The coolant flow rate, air flow rate and air pressure used in this work were 30 mL/h, 6 L/min, and 5 bar, respectively.

### 2.3. Preparation of Nanofluids

The current work involves the application of three types of commercially available nanoparticles having an average size of 40 nm. These nanoparticles are aluminum oxide (Al_2_O_3_), molybdenum disulfide (MoS_2_), and graphite respectively. The 3 wt. % nanoparticles were mixed in vegetable base oil as additives, i.e., in sunflower oil having the following physical properties: kinematic viscosity 40 1C (cSt): 40.05; viscosity index: 206; flashpoint (0 °C): 252; and pour point (0 °C): –12.00. For proper mixing of nanoparticles with base oil, the two-step method was adopted. In this method, sonication was carried out with the help of an ultra-sonication bath for about one hour followed by hot magnetic stirring of half an hour. In order to enhance the dispersion, reduce surface tension, and improve wettability and oxidation resistance, sodium lauryl sulfate and the natural antioxidant tocopherol (vitamin E) were used as surfactant at a ratio of 1:10. The properties of different nanofluids are shown in Table 4.

### 2.4. Machining Characteristic Measurements

In this work, three prominent machining indices, namely main cutting forces (Fc), tool wear (*VB_max_*), and surface roughness values (Ra) were evaluated under three different nanofluid cutting conditions. The main cutting forces were measured using online mode and the tool wear as well as surface roughness measured using offline mode. For the measurement of cutting forces, the TeLC made lathe tool dynamometer associated with XKM 2000 software was used. In same context, the tool flank wear measurements for the finish turning operation were recorded using a standard Mitutoyo’s make toolmaker’s microscope (i.e., *VB_max_* ≥ 0.60 mm, as per the ISO 3685 standard). Similarly, the arithmetic roughness values have been measured with the Mitutoyo make SJ301 surface roughness tester. Moreover, these conditions were considered for evaluation of surface roughness values, i.e., standard ISO 1999 profile R cut off length of 0.8 mm, range—auto, and speed of 0.25 mm/s. In the end, the tool wear was analyzed with the help of scanning electron microscopy (SEM).

### 2.5. Process Parameters and Design Methodology 

**Figure 1:**
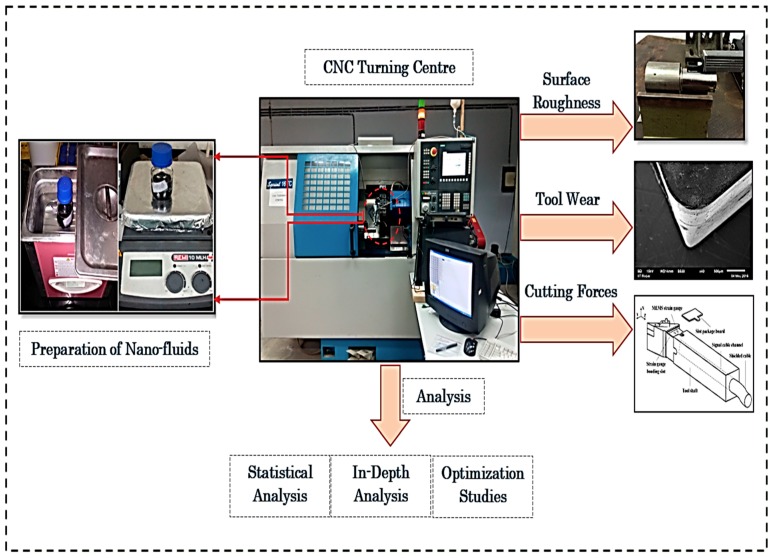
Research methodology of current work.

The three types of machining parameters with three different levels, namely cutting speed, feed rate, and depth of cut have been used in this work. The complete details of parameters and their used levels are detailed in Table 5. The selection of these parameters was based purely on pilot experiments, literature review, and tool manufacturer recommendations. The machining time of 1 min was fixed in all set of turning experiments. Moreover, these experiments were performed by following the Box-Behnken response surface methodology (RSM) design. In this, the machining parameters are considered as a continuous factor and different cooling conditions are termed as a categorical factor. Note that the total 29 experiments was suggested by RSM and for each set of experiments, a fresh cutting edge of CBN tool has been used to accurately study the effect of process parameters on machining responses. In the end, the given parameters were optimized by composite desirability approach (CDA). The main aim of the implementation of CDA is to achieve the most accurate predictions and results in the minimum possible time. The complete methodology of this scientific work is shown in Figure 1.

## 3. Results and Discussion

This section is divided into three phases: (1) Statistical Analysis, (2) Experimental Investigation of Process Parameters and (3) Optimization Studies. The complete details are given in the following sections.

### 3.1. Statistical Analysis

Statistical relevance was determined for the tested parameters. Therefore, the Box Cox transformation was developed to train the predictive models. It can help to build a family of transformations that can contains normalized data. They are normally unevenly distributed and linked to an appropriate exponent (lambda, l). By using the lambda value, it is possible to easily control the power in order to modify these data. Initially, the Box and Cox were applied to simultaneously correct the normality, linearity, and homogeneity. 

**Figure 2:**
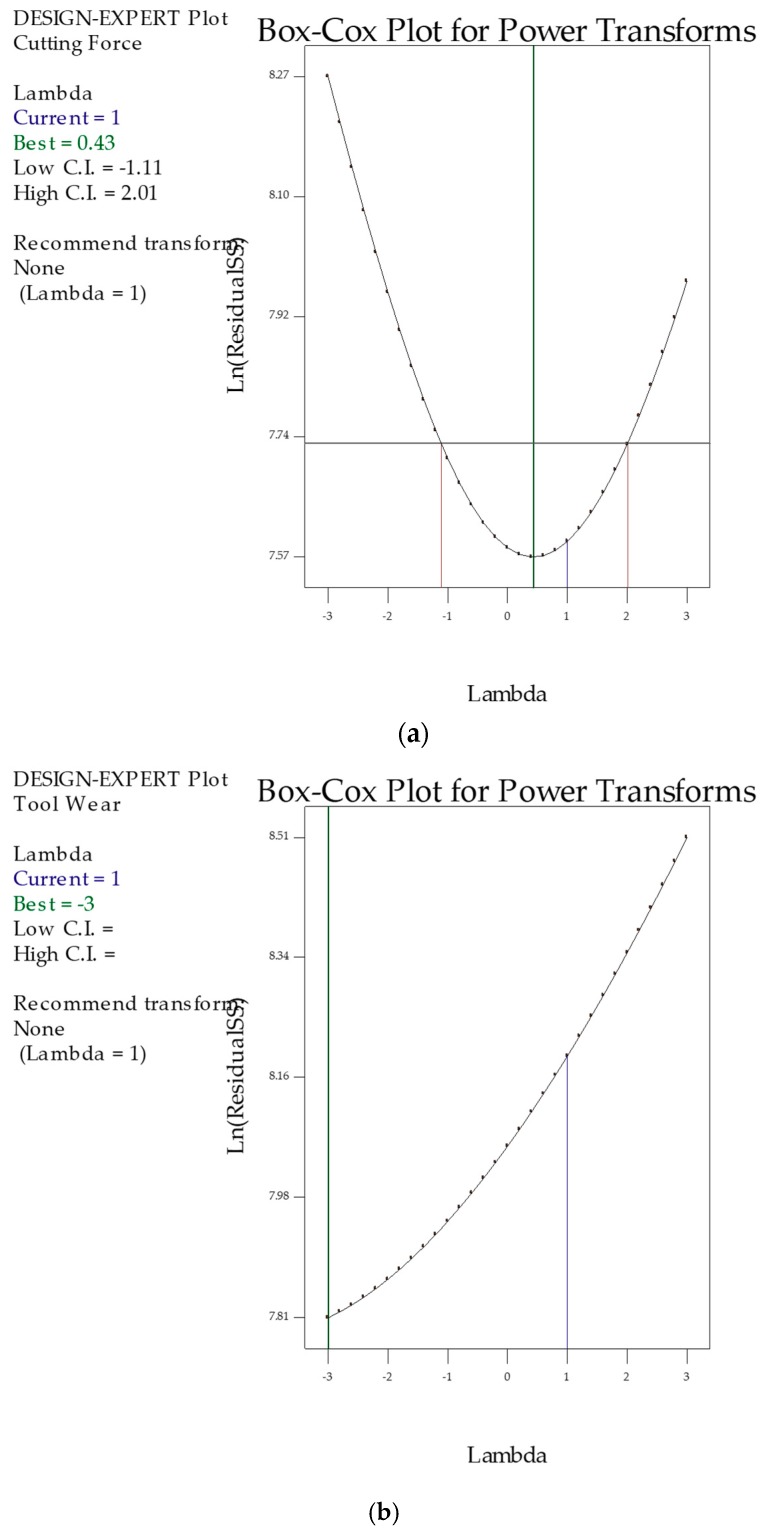

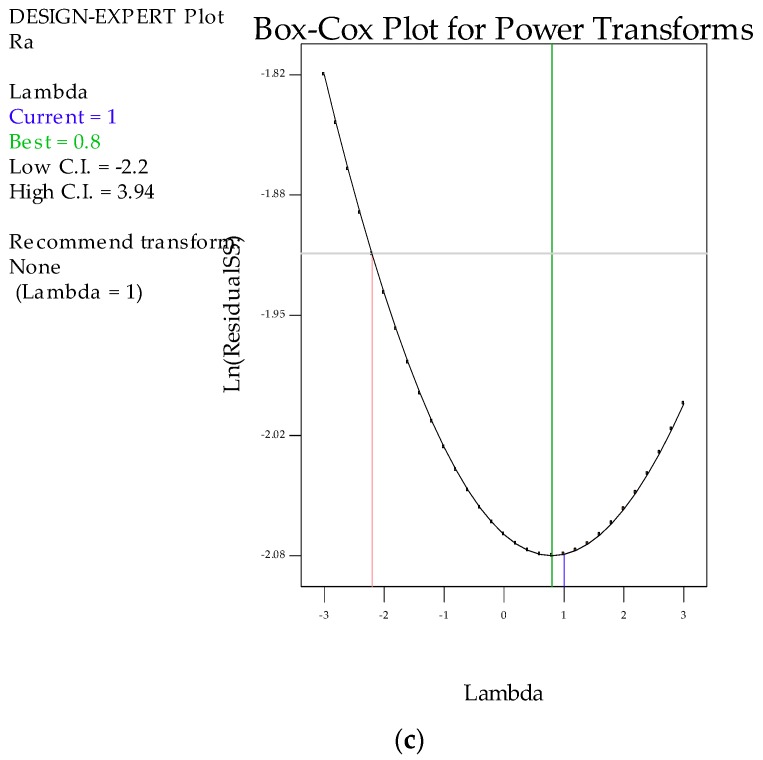
Box-Cox plots. (**a**) Cutting forces; (**b**) Tool wear; (**c**) Surface roughness.

**Figure 3:**
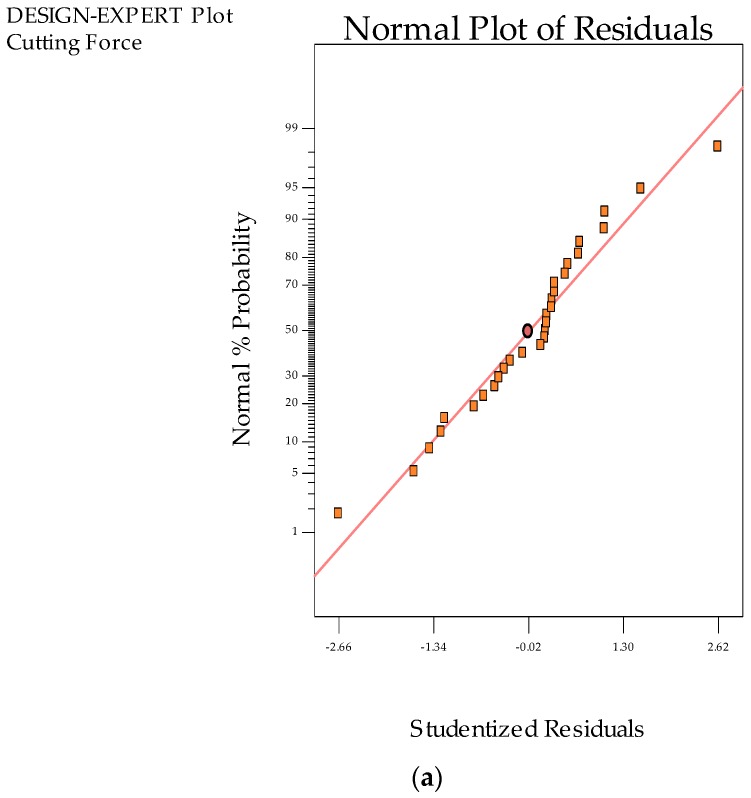

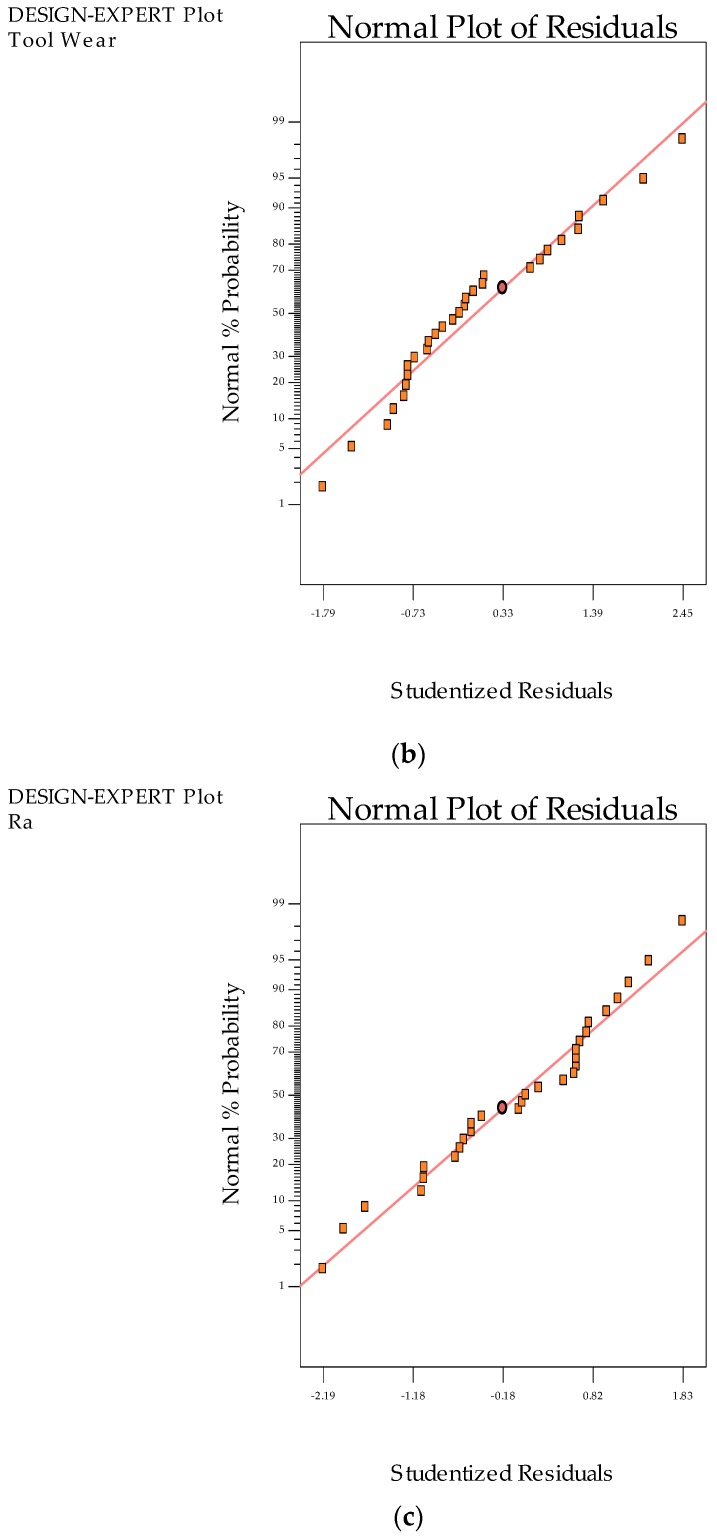
Normal distribution plots. (**a**) Cutting forces; (**b**) Tool wear; (**c**) Surface roughness.

The Box Cox plots associated with the cutting forces, surface roughness, and tool wear was presented in Figure 2a–c. In all responses, the blue line obtained from the cutting forces and surface roughness shows a value for lambda residuals equal to 1, having values outside of the 95% confidence limits. As per the green line observation, the lambda is approx. 0.5. The square root transformation is applied to the responses, which allows for the generation of normally distributed residuals. The Box Cox transformation results for the normal distribution plot of residuals are depicted in Figure 3a–c. We can note that the residuals fall over the straight line conveying the evolution of residuals were distributed as normal. Finally, the ANOVA was introduced for verification. 

The values of manual elimination procedure for the cutting force, surface roughness, and tool wear determined by ANOVA were introduced. The data from these tables demonstrates that the simulated models for all the responses are significant. There were noted the value of “Probability> F’” that is associated with lack of fit ~0.0124 (cutting forces), 0.0431 (tool wear), and 0.0173 (surface roughness). As it is larger than 0.05, the lack of fit is considered insignificant. The R^2^ values are 0.918 (cutting forces), 0.742 (tool wear), and 0.7173 (surface roughness). The measure of proportion for the entire variability, R2, helps to explain the model and was found to equal or be close to 1, as per recommendations [27]. Further, the adjusted R^2^ value was found good agreement that aided in comparing the models if they had a different number of terms. Our simulation indicates a close match between the adjusted and predicted R^2^ value. Finally, the Equations (1) to (3) were used to determine the final regression model used to determine the output parameters (i.e., cutting forces, tool wear, and surface roughness) when used different nano-cutting fluids as coded factors:

**Main Cutting Force**

(1a) $$\begin{array}{l} Cutting\;Fluid\;1:\\ \mathrm{Cutting}\;\mathrm{Force}=-58.33+0.645\times \;\mathrm{Cutting}\;\mathrm{Speed}+521.66\times \mathrm{Feed}\;\mathrm{Rate}+0.356\times \mathrm{Depth}\;\mathrm{of}\;\mathrm{Cut} \end{array}$$

(1b) $$\begin{array}{l} Cutting\;Fluid\;2:\\ \mathrm{Cutting}\;\mathrm{Force}=-70.28+0.983\times \mathrm{Cutting}\;\mathrm{Speed}+324.61\times \mathrm{Feed}\;\mathrm{Rate}+0.74\times \mathrm{Depth}\;\mathrm{of}\;\mathrm{Cut} \end{array}$$

(1c) $$\begin{array}{l} Cutting\;Fluid\;3:\\ \mathrm{Cutting}\;\mathrm{Force}=-79.66+0.85\times \mathrm{Cutting}\;\mathrm{Speed}+642.74\times \mathrm{Feed}\;\mathrm{Rate}+0.42\times \mathrm{Depth}\;\mathrm{of}\;\mathrm{Cut} \end{array}$$

**Tool Wear-**

(2a) $$\begin{array}{l} Cutting\;Fluid\;1:\\ \mathrm{Tool}\;\mathrm{Wear}=73.33+0.485\times \mathrm{Cutting}\;\mathrm{Speed}+291.66\times \mathrm{Feed}\;\mathrm{Rate}-0.144\times \mathrm{Depth}\;\mathrm{of}\;\mathrm{Cut} \end{array}$$

(2b) $$\begin{array}{l} Cutting\;Fluid\;2:\\ \mathrm{Tool}\;\mathrm{Wear}=67.36+0.647\times \mathrm{Cutting}\;\mathrm{Speed}+472.34\times \mathrm{Feed}\;\mathrm{Rate}-0.248\times \mathrm{Depth}\;\mathrm{of}\;\mathrm{Cut} \end{array}$$

(2c) $$\begin{array}{l} Cutting\;Fluid\;3:\\ \mathrm{Tool}\;\mathrm{Wear}=59+0.32\times \;\mathrm{Cutting}\;\mathrm{Speed}+542.62\times \mathrm{Feed}\;\mathrm{Rate}-0.376\times \mathrm{Depth}\;\mathrm{of}\;\mathrm{Cut} \end{array}$$

**Surface Roughness**

(3a) $$\begin{array}{l} Cutting\;Fluid\;1:\\ \mathrm{Surface}\;\mathrm{Roughness}=0.5+1.82\times 10^{-3}\times \mathrm{Cutting}\;\mathrm{Speed}+2.466\times \mathrm{Feed}\;\mathrm{Rate}-{1.94\times 10}^{-3}\times \;\mathrm{Depth}\;\mathrm{of}\;\mathrm{Cut} \end{array}$$

(3b) $$\begin{array}{l} Cutting\;Fluid\;2:\\ \mathrm{Surface}\;\mathrm{Roughness}=0.42578+{1.2354\times 10}^{-3}\times \;\mathrm{Cutting}\;\mathrm{Speed}+5.42367\times \mathrm{Feed}\;\mathrm{Rate}-{1.424\times 10}^{-3}\times \;\mathrm{Depth}\;\mathrm{of}\;\mathrm{Cut} \end{array}$$

(3c) $$\begin{array}{l} Cutting\;Fluid\;3:\\ \mathrm{Surface}\;\mathrm{Roughness}=0.45+1{.897\times 10}^{-3}\times \;\mathrm{Cutting}\;\mathrm{Speed}+7.336\times \mathrm{Feed}\;\mathrm{Rate}-1.798\times 10^{-3}\times \mathrm{Depth}\;\mathrm{of}\;\mathrm{Cut} \end{array}$$

### 3.2. Experimental Investigation

#### 3.2.1. Cutting Forces

**Figure 4:**
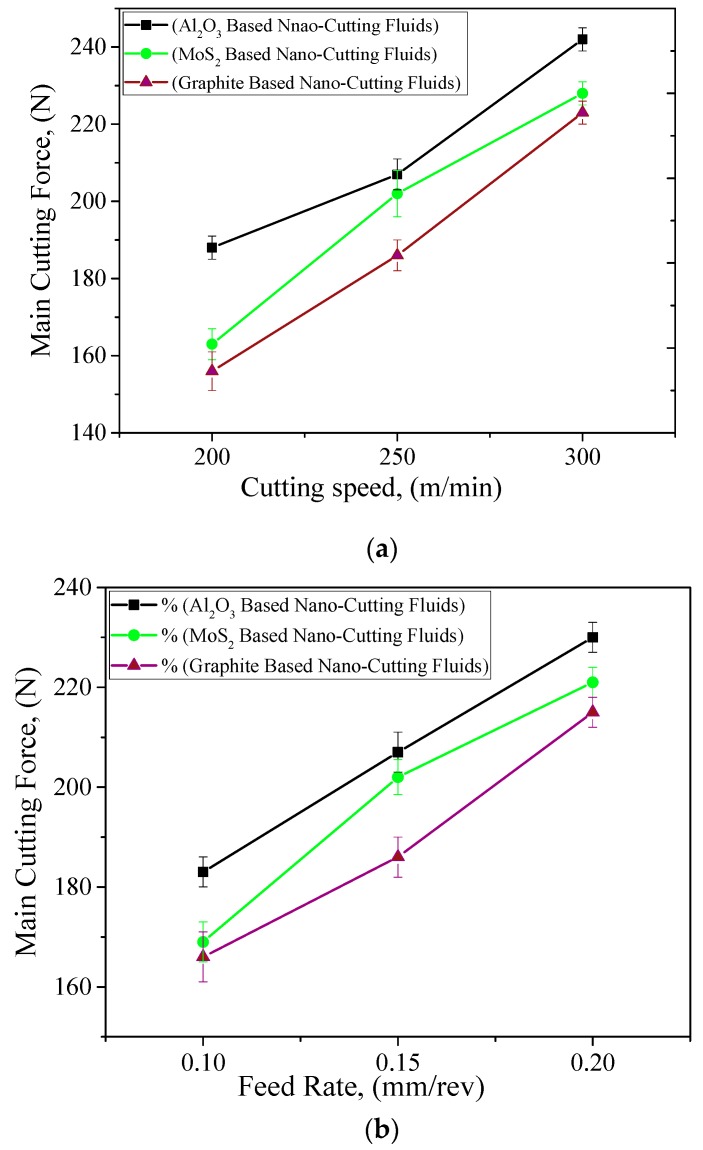

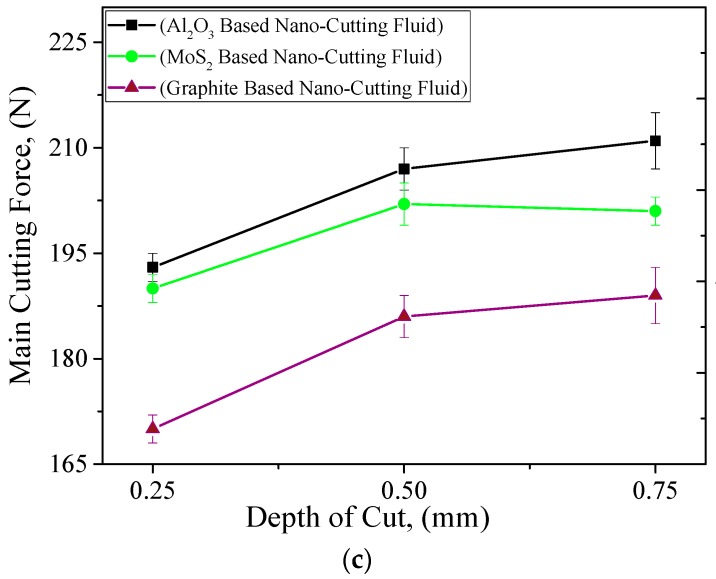
Effect of cutting parameters on main cutting forces. (**a**) Cutting Speed, where *f* = 0.15 mm/rev *&* *a_p_* = 0.50 mm; (**b**) Feed Rate, where *Vc* = 250 mm/rev and *a_p_* = 0.50 mm; (**c**) Depth of Cut, where *Vc* = 250 mm/rev and *f* = 0.15 mm/rev.

The cutting forces play an important role in the deformation of machine tool structure. However, they are directly associated with the machine tool dynamics, and the main factors which affect the cutting forces are cutting speed, feed rate, and depth of cut. Moreover, the application of cooling conditions significantly helps to reduce the cutting forces during turning operation, and this phenomenon becomes more prominent when the nanofluids are used along with the MQL system. Therefore, the main cutting force is considered as the important machining index in this experimental work. The effect of machining parameters and cooling conditions on main cutting force is shown in Figure 4a–c. It is clearly noted that the cutting forces are increased with the increase in cutting speed and feed rate values. However, the change in depth of cut produces very little variations in the main cutting force values. The increase in cutting speed may have increased the temperature which caused local work hardening. Consequently, a higher force is required for material deformation. The increase of force in cutting with increased feed rate is due to an increase in the chip cross-section, i.e., more friction [28]. In other words, the cutting speed and feed rate are the most significant factors that contribute in increasing the main cutting force values as compared with the depth of cut. The same results are also calculated using the ANOVA tests. Similarly, the trend of cutting environment are described in these figures. It is highly visible that the cutting forces are significantly reduced with the application of change in cutting fluid from Al_2_O_3_-based nanofluids to graphite-based nanofluids and MoS_2_-based nano-cutting fluid shows a moderate effect on the main cutting force values. This is justified with the two properties of cutting fluids presented in Table 4: (1) viscosity and (2) thermal conductivity. It should be stated that using graphite-based nanofluid enhances the heat transfer and tribological performance of the cutting process. The applied nano-mist with compressed air shows capabilities in penetrating into the tool–workpiece interface area, and reduces the severity of the induced heat during machining operation. In addition, the employed nano-mist acts like rollers and causes a noticeable effect in the frictional behavior of the cutting operation, as has been discussed in the literature [29,30,31].

#### 3.2.2. Tool Wear

**Figure 5:**
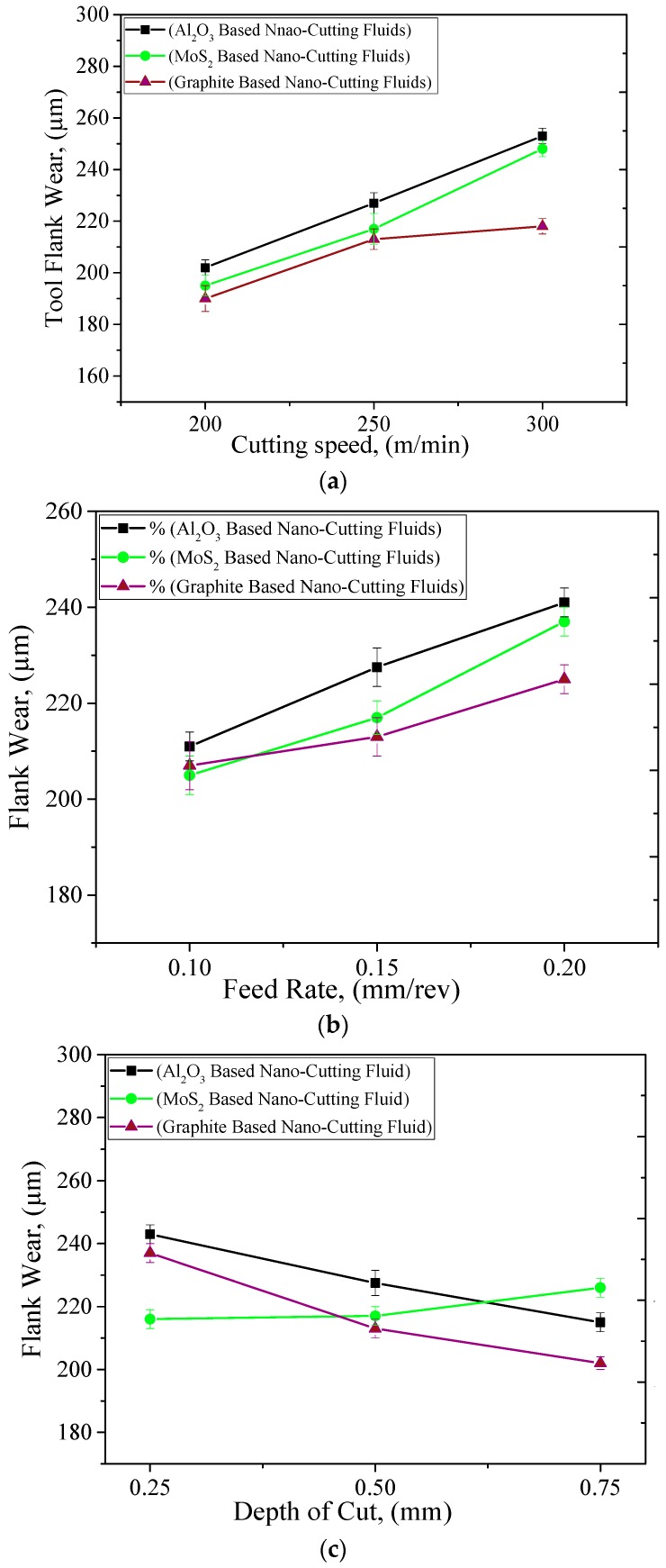
Effect of cutting parameters on tool wear. (**a**) Cutting Speed, where *f* = 0.15 mm/rev *&* *a_p_* = 0.50 mm; (**b**) Feed Rate, where *Vc* = 250 mm/rev and *a_p_* = 0.50 mm; (**c**) Depth of Cut, where *Vc* = 250 mm/rev and *f* = 0.15 mm/rev.

**Figure 6:**
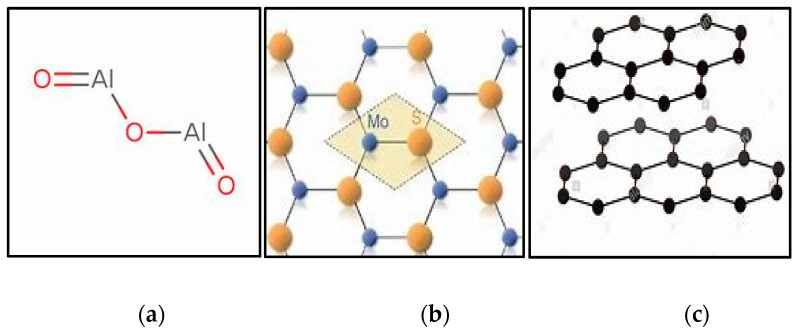
Atomic structure of the used nanoadditives. (**a**) Alumina structure; (**b**) MoS_2_ structure; (**c**) Graphite structure.

**Figure 7:**
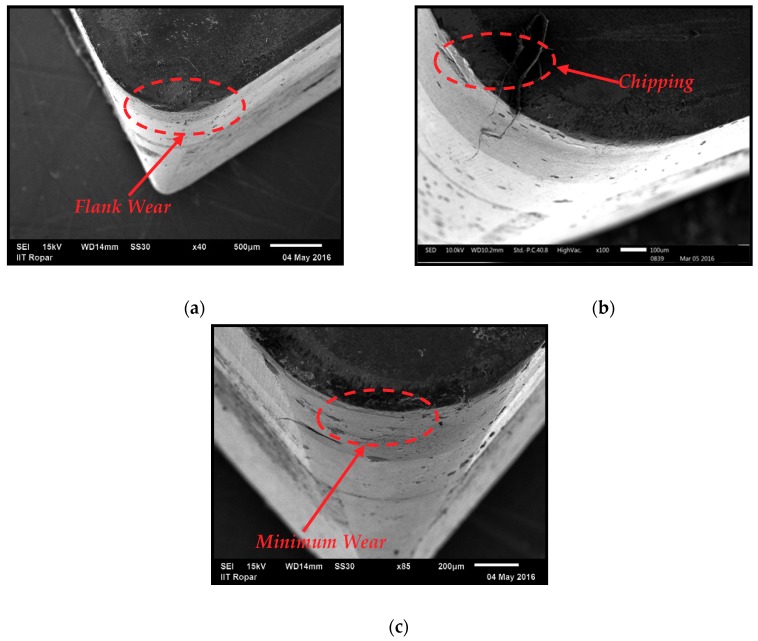
Tool wear images at different working conditions at *V_c_* = 300 m/min, *f* = 0.15 mm/rev, *a_p_* = 0.50 mm. (**a**) Al_2_O_3_ nanofluid; (**b**) MoS_2_ nanofluid; (**c**) Graphite nanofluid.

The optimum machining cost and minimum energy is achieved with the tool having longer life because the tool wear and tool life are critically damaged with the direct values of machining parameters. Therefore, the careful selection of cutting speed, feed rate, and depth of cut values are required to achieve the maximum tool life values. In addition, the presence of coolant/lubricant has cemented their potential to improve the tool life values. Therefore, in this work, the maximum flank wear criteria according to the ISO 3685 (i.e., VB_max_ ≥ 400 µm) has been selected to evaluate the results. The influence of cutting speed, feed rate, and depth of cut under the different nanofluids conditions are presented in Figure 5a–c. It is noted that for cutting speed and feed rate values, the tool flank wear follows an increasing pattern. However, the depth of cut shows the opposite trend and very little effect on tool flank wear values as compared with the cutting speed followed by the feed rate values. As mentioned earlier, the increased chip contact area and the hardening effect at local zone may have caused the difficulty in cutting. As such, the wear of tool increased. Furthermore, it is observable that the values of tool flank wear were reduced with the different cutting fluids values and the graphite-based nano-cutting fluids shows promising results. In terms of the tool performance, the nano-mist from all nano-additives showed promising performance in reducing the severity of the tool wear, and this is mainly due to the enhancement in the rubbing level (i.e., at the tool–workpiece interface zone). As mentioned earlier, the nano-mist acts as rollers, and that reduces the induced coefficient of friction. As can be seen in Figure 5, graphite-based nano-mist offers less flank wear than Al_2_O_3_ and MoS_2_ nano-mist. This can be due to the higher thermal conductivity (see Table 4), which means better heat transfer and less rubbing effects. In addition, the giant covalent structure in graphite atomic structure can lead to a better rolling effect, especially when it is compared to the alumina atomic structure (see Figure 6). That can be reflected in the results obtained in Figure 5 as the graphite and MoS_2_ nano-mist offer almost the same performance with a slight advantage over the graphite nanofluid. Furthermore, noticeable effects of both graphite and MoS_2_ nano-mist clearly appeared at high cutting speeds (see Figure 5a) which mean that a high heat was generated at the cutting zone. Moreover, the SEM images in Figure 7 confirmed the same findings in Figure 5 as all used nano-additives offered better tool performance; however, greater advantages have been observed for both graphite and MoS_2_ nano-mist, especially in crater wear. 

### 3.3. Surface Roughness

**Figure 8:**
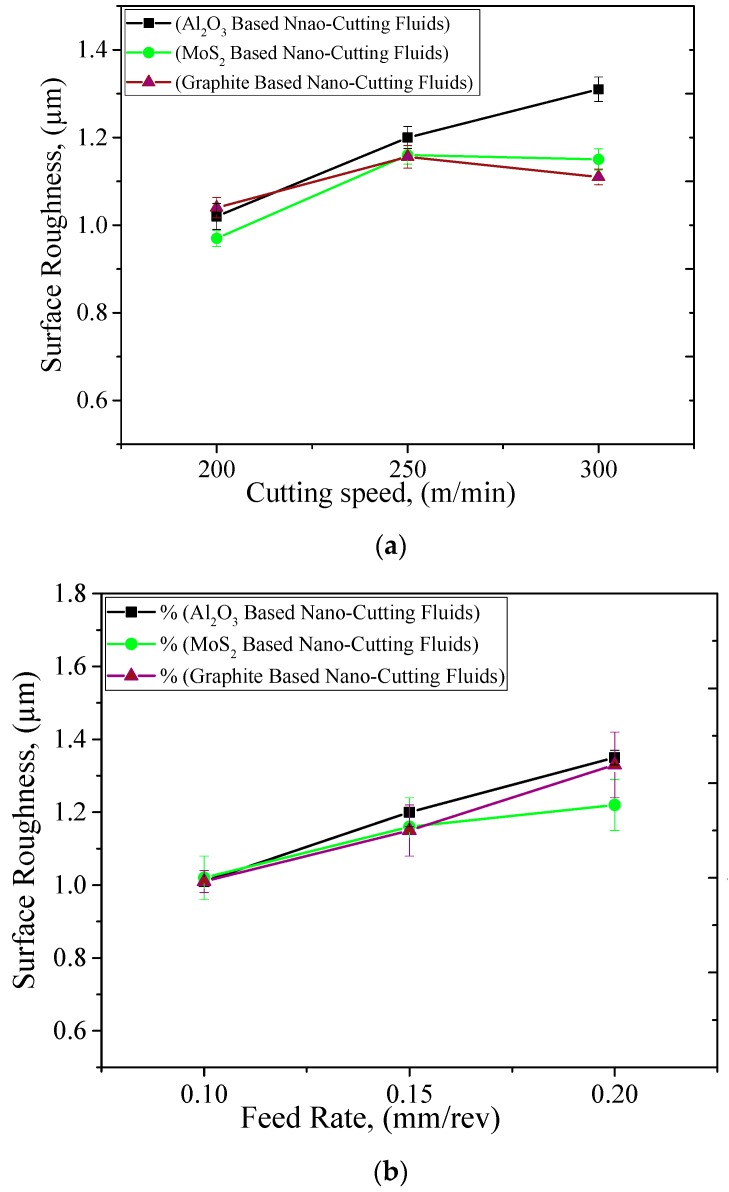

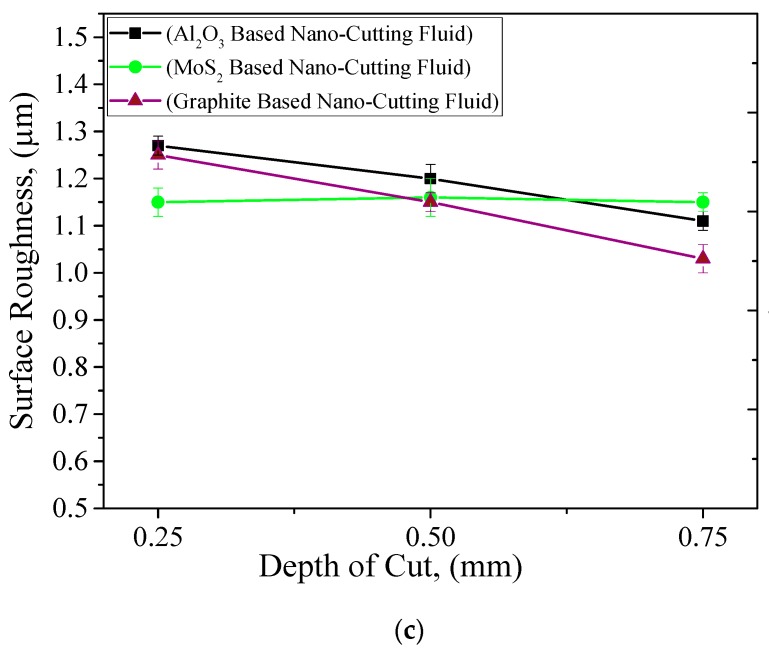
Effect of cutting parameters on surface roughness. (**a**) Cutting Speed, where *f* = 0.15 mm/rev *&* *a_p_* = 0.50 mm; (**b**) Feed Rate, where *Vc* = 250 mm/rev and *a_p_* = 0.50 mm; (**c**) Depth of Cut, where *Vc* = 250 mm/rev and *f* = 0.15 mm/rev.

In order to evaluate the quality of any product, arithmetic surface roughness (Ra) is considered as a valuable parameter. As evident from Figure 8, the increase in the cutting speed caused an increment in surface roughness. This can be attributed to the increased chatter of the machine tool at such a high cutting speed (300 m/min) used for a superalloy. Similarly, the increase in feed rate caused an increase in the area of tool travel; in other words, more friction was endured. As a result, the tool wear increased. Higher friction and tool wear may have caused the surface roughness to be increased at such a higher feed rate. However, the increase in depth of cut has caused the surface roughness to be lower. Here, it is to be noted that the impingement of cooling agent with nanofluid have caused the surface to be smoother and a lower roughness value was found. It can be noted that the performance of the three used nanoadditives were nearly the same; however, both graphite and MoS_2_ nano-mist showed better results at the highest cutting speed (see Figure 8). As mentioned earlier, this can be due to the higher thermal conductivity compared to Al_2_O_3_ nanofluid. This enhancement results from the promising heat transfer and tribological performance of such nanoadditives, which improve the interactional effect between the tool and workpiece, and reduce the induced coefficient of friction as well as the high generated temperature in the cutting zone. Therefore, better tool performance and surface quality can be clearly observed compared to the classical technique, as previously discussed in some previous studies [32,33].

In order to physically understand the provided results, the nano-mist mechanism during cutting processes should be discussed in terms of the tribological and heat transfer aspects. Due to the effect of the MQL compressed air along with the nanofluids, a very thin layer is formed at the cutting zone as described in the literature [34,35]. This layer includes two main advantages to the overall performance of the cutting process. The first advantage is to absorb the high heat generated during the process as it has a high heat transfer coefficient because of the employed nanoadditives [36]. The second aspect is related to the friction behavior. The applied nano-mist at cutting zone plays as rollers which reduce the induced rubbing between the tool and workpiece [37]. Therefore, lower cutting forces and tool wear can be observed for the cutting tests performed with MQL nanofluid. 

### 3.4. Optimization of Process Parameters: Composite Desirability Approach (CDA)

The main objective was to identify the best possible process parameters that lead to sustainable machining of Inconel-800 alloy. Table 6 presents the results obtained using the typical desirability strategy. This approach is very common and provides an efficient solution with a friendly interface [8,9]. It is developed to adjust the characteristic weight and their importance. Through this approach, it is possible to combine all assigned goals to a unique desirable function in the range $0\leq d_{i}\leq 1$. Details of this approach were presented elsewhere [38]. The outputs responses determined through this approach are classified as (1) higher-the-better, (2) smaller-the-better, and (3) nominal-the-better. To ensure the goal of this research, here, we implemented ‘smaller-the-better’ responses that were evaluated numerically through Equation (4).

(4) $$d_{i}=\left\{\begin{array}{cc} 1 & x_{i}\leq x_{1}^{"}\\ {\left[\frac{x_{i}^{*}-x_{i}}{x_{1}^{*}-x_{1}^{"}}\right]}^{r} & x_{1}^{"}<x_{i}<x_{i}^{*}\\ 0, & x_{i}\geq x_{i}^{*} \end{array}\right\}$$

Here, $x_{1}^{"}$ represents the smallest value associated to $x_{i}$, $x_{1}^{*}$ is the largest value associated to $x_{i}$ while *r* denotes the shape function.

**Figure 9:**
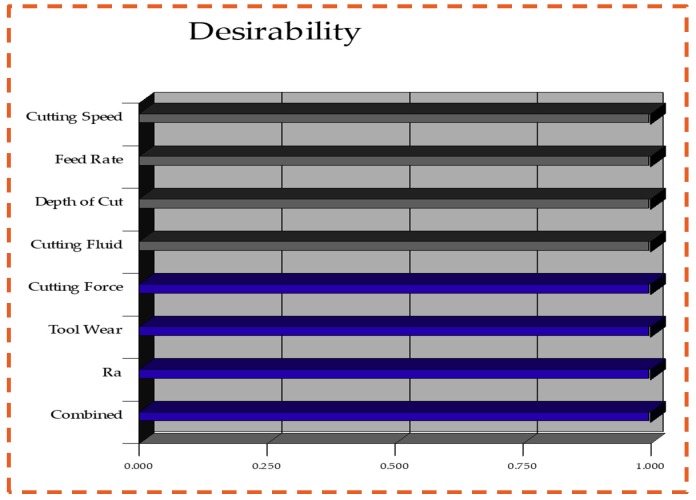
Histogram plot represent the optimum values.

Further, the cutting speed, feed rate, and depth of cut were selected within the range parameters. Table 6 presents the optimum five solutions determined by applying the CDA. The best solution was considered as the one having the maximum desirability value. Similarly, Figure 9 shows the histogram determined for the ideal solution with following parameters: 200 m/min for the cutting speed, 0.10 mm/rev of feed rate, 0.70 mm of depth of cut, and graphite-based nano-cutting fluids having the maximum desirability value. 

## 4. Conclusions

This work primarily focuses on the performance of three different nano-cutting fluids during the turning of new nickel-based alloy, i.e., Inconel-800. In the literature, major efforts have been focused on the Inconel-718 alloy. That is why this new alloy has been selected as a subject material in this experimental work. The major conclusions drawn from these experiments are given below:

- Statistical analysis results: The results determined through experiments were statistically significant in terms of Box Cox transformation, R^2^ values, and ANOVA tests. Therefore, the prediction models are useful for researchers and academics to determine the values for their reference.
- Experimental investigation: The trend of almost all parameters were found to be the same, i.e., the cutting forces, tool wear, and surface roughness values were significantly affected with small changes in any one of these machining parameters.
- Comparison results: When the comparison was made between all cutting fluids, the overall performance of graphite-based nanofluids was found to be better in improving the machining characteristics. This is because of the good tribological and cooling properties of graphite-based nano-cutting fluids. Moreover, the chemical structure of graphite is more covalent and this drastically affects its performance as compared to other nanofluids.
- Optimization results: CDA is also a very efficient optimization method for determining the optimal solution, i.e., 200 m/min for the cutting speed, 0.10 mm/rev of feed rate, 0.70 mm of depth of cut, and graphite-based nano-cutting fluids.
- Future recommendations: Even though the results obtained from this study were highly useful for practical applications, some future avenues are still pending to improve the machining performance of Inconel-800 alloy. For instance, the high-pressure cooling (HPC) approach could be integrated with the nano-cutting fluids and the results compared with the MQL technique.

## Figures and Tables

**Table 1:** Chemical composition of Inconel-800 alloy.

Ni	Cr	Fe	C	Al	Ti	Al + Ti
30.0–35.0	19.0–23.0	39.5 min	0.10 max	0.15–0.60	0.15–0.60	0.30–1.20

**Table 2:** Heat treatment conditions of Inconel-800 super alloy.

Heat Treatment	Intermediate Treatment	Final Treatment	Rockwell Hardness
1050 °C for 2 h, air-cooling	850 °C for 6 h, air-cooling	700 °C for 2 h, air-cooling	RC

**Table 3:** Tool geometry of cutting tool.

Inclination Angle	−6°
Orthogonal rake angle	6°
Orthogonal clearance angle	80°
Auxiliary cutting-edge angle	15°
Principal cutting-edge angle	90°
Nose radius	0.4 mm
Shape	Rhombic

**Table 4:** Properties of different nanofluids [26].

Properties	Vegetable Base Oil	Al_2_O_3_ Nanofluid	MoS_2_ Nanofluid	Graphite Nanofluid
Appearance	Bright and clear	White	Black	Grayish Black
Viscosity (CP) (at 20 °C)	68.16	120.23	100.56	83.12
Thermal Conductivity (W/mK)	0.1432	0.2085	0.2362	0.2663

**Table 5:** Machining parameters and their levels.

Parameters	Coded Value	Units	Low Level (−1)	Middle Level (0)	High Level (+1)
Cutting Speed (Vc)	A	m/min	200	250	300
Feed Rate (f)	B	mm/rev	0.1	0.15	0.20
Depth of cut (*a_p_*)	C	mm	0.25	0.50	0.75
Cooling condition	D	-	Al_2_O_3_	MoS_2_	Graphite

**Table 6:** Optimum results using composite desirability approach (CDA).

Sr. No.	Cutting Speed	Feed Rate	Depth of Cut	Cutting Fluid	Cutting Force	Tool Wear	Surface Roughness	Desirability
1	200	0.10	0.70	3	143	181	0.87	1.00
2	202	0.10	0.64	3	141	183	0.88	0.88
3	201	0.10	0.63	3	140	183	0.88	0.74
4	201	0.10	0.70	3	145	182	0.87	0.72
5	200	0.10	0.62	2	141	182	0.88	0.65
